# WSES project on decision support systems based on artificial neural networks in emergency surgery

**DOI:** 10.1186/s13017-021-00394-9

**Published:** 2021-09-26

**Authors:** Andrey Litvin, Sergey Korenev, Sophiya Rumovskaya, Massimo Sartelli, Gianluca Baiocchi, Walter L. Biffl, Federico Coccolini, Salomone Di Saverio, Michael Denis Kelly, Yoram Kluger, Ari Leppäniemi, Michael Sugrue, Fausto Catena

**Affiliations:** 1 Department of Surgical Disciplines, Immanuel Kant Baltic Federal University, Kaliningrad, Russia; 2 Kaliningrad Branch of Federal Research Center “Computer Science and Control” of Russian Academy of Sciences, Kaliningrad, Russia; 3 Department of Surgery, Macerata Hospital, Macerata, Italy; 4 Surgical Clinic, Department of Experimental and Clinical Sciences, University of Brescia, Brescia, Italy; 5 Division of Trauma and Acute Care Surgery, Scripps Memorial Hospital La Jolla, La Jolla, CA USA; 6 General, Emergency and Trauma Surgery Department, Pisa University Hospital, Pisa, Italy; 7 Department of Surgery, Cambridge University Hospital, NHS Foundation Trust, Cambridge, UK; 8 Department of General Surgery, Albury Hospital, Albury, Australia; 9 Department of General Surgery, Rambam Healthcare Campus, Haifa, Israel; 10 Department of Gastrointestinal Surgery, University of Helsinki and Helsinki University Hospital, Helsinki, Finland; 11 Donegal Clinical Research Academy, Letterkenny University Hospital, Donegal, Ireland; 12 Department of Emergency and Trauma Surgery of the University Hospital of Parma, Parma, Italy

**Keywords:** Decision support system, Artificial neural networks, Emergency surgery, Acute appendicitis, Acute pancreatitis, Acute cholecystitis, Bowel obstruction, Perforated gastroduodenal ulcers, Peptic ulcer bleeding, Strangulated hernias

## Abstract

The article is a scoping review of the literature on the use of decision support systems based on artificial neural networks in emergency surgery. The authors present modern literature data on the effectiveness of artificial neural networks for predicting, diagnosing and treating abdominal emergency conditions: acute appendicitis, acute pancreatitis, acute cholecystitis, perforated gastric or duodenal ulcer, acute intestinal obstruction, and strangulated hernia. The intelligent systems developed at present allow a surgeon in an emergency setting, not only to check his own diagnostic and prognostic assumptions, but also to use artificial intelligence in complex urgent clinical cases. The authors summarize the main limitations for the implementation of artificial neural networks in surgery and medicine in general. These limitations are the lack of transparency in the decision-making process; insufficient quality educational medical data; lack of qualified personnel; high cost of projects; and the complexity of secure storage of medical information data. The development and implementation of decision support systems based on artificial neural networks is a promising direction for improving the forecasting, diagnosis and treatment of emergency surgical diseases and their complications.

## Introduction

Currently, computer decision support systems (DSS) based on algorithms developed with the use of the methods of intellectual analysis of medical data are receiving special development [1–3]. It is known [3] that when making medical decisions, there are such problems as lack of knowledge, limited time resources, inability to attract a large number of competent experts, incomplete information about the patient's condition, etc. There is an urgent need to use different methods of computer decision support in emergency surgery wider [4]. Decision support systems can enable a surgeon to check his own prognostic and diagnostic assumptions in emergency and to use artificial intelligence in complex urgent clinical cases [5, 6]. DSS frequently are developed based on artificial neural networks (ANNs) [7–9].

ANNs are mathematical models that allow, as a rule, the classification of objects somewhat better than probabilistic (stochastic) models [10]. ANNs are based on some principles of information processing in biological systems. ANN consists, in most cases, of relatively simple, elements that imitate the function of brain neurons. Each artificial neuron is analogous with nerve cells in the brain, which can be fired or inhibited. The artificial neuron receives one or more inputs (representing excitatory postsynaptic potentials and inhibitory postsynaptic potentials at neural dendrites) and sums them to produce an output (or activation, representing a neuron's action potential which is transmitted along its axon) [11]. ANNs are characterized by the principle of parallel signal processing, which is achieved by combining a large number of neurons into so-called layers. Neurons of different layers are connected in a specific way. The strength of synaptic links is modified in the process of extracting knowledge from the training dataset (training mode), and then used when obtaining a result on new data (execution mode) [10]. Neural networks make decisions based on hidden patterns they reveal in multidimensional data.

Neural networks are self-learning in distinction to classical machine learning. There is a deep learning in neural networks, which is the most popular and high-demand because an amount of data and computing power are constantly growing [12]. It is considered that the complexity of the neural network should correspond to the complexity of the phenomenon under study. Moreover, in many cases, very simple neural networks with two layers are even more accurate than very deep networks with many layers. The reasons for this [13] may lie in the insufficient amount of data, in the excessive complexity of the network in comparison with the phenomenon under study, etc. In general, the trend is that neural networks are increasingly replacing classical machine learning, and deep learning prevails over simple neural networks with a small number of layers [12].

Neural networks have found applications in many fields of medicine for solving multiple applied problems [14–18]. Examples of the use of computer prediction based on ANN technology in surgical specialties are also quite numerous [19–21].

The aim of the article is to scoping review the literature on the effectiveness of ANN-based DSS for the diagnosis, prognosis and treatment of urgent surgical diseases.

## Methods

A scoping review was conducted following the guidelines of PRISMA-ScR (Preferred Reporting Items for Systematic Reviews and Meta-Analyses Extension for Scoping Reviews) [22]. We assessed electronic database PubMed. The following search criteria have been used: “artificial neural networks” AND “acute appendicitis,” “artificial neural networks” AND “acute pancreatitis,” “artificial neural networks” AND “acute cholecystitis,” “artificial neural networks” AND “bowel obstruction,” “artificial neural networks” AND “perforated gastroduodenal ulcers,” “artificial neural networks” AND “ulcer bleeding,” “artificial neural networks” AND “strangulated hernias.”

Results were reviewed based on predefined inclusion and exclusion criteria. We reviewed all peer reviewed published papers considering the use of ANNs in emergency surgery (acute appendicitis, acute pancreatitis, acute cholecystitis, ileus/bowel obstruction, perforated gastroduodenal ulcers, nonvariceal upper gastrointestinal/peptic ulcer bleeding, incarcerated/strangulated hernias), except publications older than 1990-year, case reports, editorials and commentaries.

## Results

The initial search identified 61 publications in the PubMed database. Nineteen articles met the inclusion and exclusion criteria. Characteristics of each are shown in Table 1.

**Table 1:** Results of using ANNs for diagnostics and prognosis in acute surgical diseases

Authors	The training set size	Objectives of the research	Results
*Acute appendicitis (AA)*			
Yoldaş et al. [23]	156	AA diagnostics	Sensitivity—100%, specificity—97.2%
Park and Kim [24]	801	AA diagnostics	The ANN proved to be more accurate in diagnosing AA (the accuracy of the three types of ANN—99.8%, 99.4%, 97.8%) than the Alvarado clinical scoring system (72.2%)
Reismann et al. [25]	590	AA diagnostics, prediction of a complicated course of the disease in pediatrics	The ANN has allowed a significant improvement of the accuracy of diagnosis (sensitivity 93%, specificity 67%), and complicated course of AA (sensitivity 95%, specificity 33%)
Park et al. [26]	667	AA diagnostics based on CT of patients with abdominal pains	The ANN showed good and very good diagnostic indicators of AA (accuracy > 90%)
*Acute pancreatitis (AP)*			
Kazmierczak et al. [27]	254	Diagnosis of the AP by the level of pancreatic enzymes in the blood serum	Lipase level has the highest diagnostic accuracy (accuracy lipase—82%, serum amylase—76%, lipase + amylase—84%)
Pofahl et al. [28]	156	Predicting the hospitalization length	Sensitivity 75%, specificity 81% and accuracy 79%, but the ANN predictive capabilities do not differ from Ranson and APACHE II
Keogan et al. [29]	92	Predicting the hospitalization length based on CT and laboratory tests	The ANN showed the best predictive accuracy (AUC = 0.83 ± 0.05) compared to the Ranson (AUC = 0.68 ± 0.06; *P* < 0.02) and Balthazar (AUC = 0.62 ± 0.06; *P* < 0.003)
Halonen et al. [30]	234	Predicting the potential mortality	The ANN predictive capabilities (AUC = 0.847) differ from Ranson (AUC = 0.655), APACHE II (AUC = 0.817) and Glasgow (AUC = 0.536)
Mofidi et al. [31]	496	Identification of the AP severity and predicting lethal outcome	The ANN proved to be more accurate in diagnosing of the AP (ANN was more accurate than APACHE II and Glasgow in predicting: AP severity—*P* < 0.05 and *P* < 0.01 Multiple organ failure *P* < 0.05 and *P* < 0.01 Lethal outcome—*P* < 0.05 and *P* < 0.05)
Andersson et al. [32]	139	Predicting the AP severity	The ANN proved to be more accurate (AUC = 0.92) in diagnosing of the severe AP in comparison with the logistic regression (AUC = 0.84, *P* = 0.03) and APACHE II (AUC = 0.63, *P* < 0.001)
Hong et al. [33]	312	Predicting the persistent (more than 48 h) organ failure	The ANN proved to be more accurate in predicting of the persistent organ failure (AUC = 0.96 ± 0.02) in comparison with the logistic regression (AUC = 0.88 ± 0.03, *P* < 0.001) and APACHE II (AUC = 0.83 ± 0.03, *P* < 0.001)
Fei et al. [34]	152	Predicting the severe AP associated with acute lung injury	The ANN proved to be more accurate (AUC = 0.859 ± 0.048) in predicting of the acute lung injury accompanying the AP in comparison with the logistic regression (AUC = 0.701 + 0.041)
*Acute cholecystitis (AC)*			
Eldar et al. [35]	180	Predicting the conversion from laparoscopic to laparotomic access in AC	The ANN demonstrated a good predictive ability to predict the conversion from laparoscopic to laparotomic approach (100% of cases respectively, 67%—prospectively) and to determine the group of patients requiring laparotomic cholecystectomy
Vukicevic et al. [36]	303	Prediction of choledocholithiasis in patients with gallstone disease and AC	The ANN demonstrated a good predictive ability to predict the choledocholithiasis and revealed informative clinical, laboratory, and instrumental signs (sensitivity—82.3%, specificity—94.7%, accuracy—92.2%, and AUC—0.934 in the validation set)
*Ulcerative bleeding*			
Rotondano et al. [37]	2380	Predicting the fatal outcome in patients with bleeding from the upper gastrointestinal tract	The predictive ability of the ANN is better than the score Rockall’s one [38] (sensitivity—83.8% versus 71.4%, specificity—97.5% versus 52.0%, accuracy—96.8% versus 52.9%, and AUC—0.95 versus 0.67)
Wong et al. [39]	22,854	Identification of patients with a high risk of recurrent bleeding requiring surgical treatment and with a high risk of death	The ANN demonstrated AUC = 0.78, and accuracy—84.3%
*Perforated gastroduodenal ulcers*			
Søreide et al. [44]	117	Predicting the fatal outcome and determination of factors of the fatal outcome	AUC = 0.90, 0.95% CI [0.85–0.95]
*Ileus/bowel obstruction*			
Cheng et al. [45]	13,935 X-ray pictures	Ileus diagnostics	Sensitivity—91.4%, specificity—91.9%
*Strangulated hernia*			
Chen et al. [46]	762	Predicting the need for bowel resection	ANN revealed eight factors that are significantly associated with the need for bowel resection

### Acute appendicitis

Yoldaş et al. [23] investigated the diagnostic capabilities of ANN in acute appendicitis (AA) in patients with pain in the right lower abdomen. Data were collected from 156 patients with suspected acute appendicitis prospectively. The sensitivity, specificity, and positive and negative predictive values of the artificial neural network were 100%, 97.2%, 96.0% and 100%, respectively. The authors concluded that ANNs can be an effective tool for accurate diagnosis of acute appendicitis and can reduce the number of “unnecessary” appendectomies [23].

Park and Kim [24] also used ANN to diagnose AA. The data of 801 patients were used to create artificial neural networks of three types: ANN with a radial basis function, multilayer ANN and ANN with a probabilistic structure. The Alvarado clinical scoring system was used for comparison. The accuracy of the three types of ANN and Alvarado was 99.8%, 99.4%, 97.8% and 72.2%, respectively; ROC AUC (Area under the ROC (receiver operating characteristic) curve)—0.998, 0.993, 0.985 and 0.633, respectively. The ANN models proposed by the authors for the diagnosis of acute appendicitis showed better results than the Alvarado clinical assessment system (*P* < 0.001).

Reismann et al. [25] examined the informativeness of artificial intelligence (AI) for the diagnosis of the AA in childhood and adolescence. This study presents a method for the automatic diagnosis of appendicitis, as well as for identifying the differences between complicated and uncomplicated AA. It is based on generally accepted clinical data and diagnostic methods. The medical records of a total of 590 patients were retrospectively analyzed using AI (473 patients with acute appendicitis according to histological findings and 117 patients with negative histological findings). The accuracy of the developed automatic method for the diagnosis of the AA was 90% (sensitivity 93%, specificity 67%), and the accuracy of correct determination of the complicated course of the disease was 51% (sensitivity 95%, specificity 33%).

Park et al. [26] investigated the possibility of a neural network algorithm for the diagnosis of the AA using CT for patients with acute abdominal pain. The diagnostic characteristics of the developed model based on CT were “very good” and amounted to more than 90%.

### Acute pancreatitis

The first attempt to predict the severity of acute pancreatitis using an ANN was undertaken by Kazmierczak et al. [27] by analyzing the serum concentration of pancreatic enzymes in blood serum. Pancreatic lipase was the best predictor of the severity of AP, with an accuracy of 82% (95% confidence interval (CI): 77–87). In contrast, using the serum amylase value, the prediction accuracy turned out to be quite low—76% (71–81). Meanwhile, the combination of such indicators as lipase and amylase did not increase the accuracy of the created ANN significantly—the predictive accuracy was 84% (79–89) [27].

Pofahl et al. [28] used ANN to predict the hospital length of stay (LOS) of patients with AP. In their study, they compared ANNs with the Ranson and APACHE II scoring systems in terms of its sensitivity, specificity, positive predictive value and accuracy. The authors provide information on the high sensitivity (75%), specificity (81%) and accuracy (79%) of ANN in identification of the acute pancreatitis severity as measured by LOS [28].

In research by Keogan et al. [29], a neural network model was used to predict a prolonged treatment duration for a patient with AP, longer than the average of 8.4 days. The ANN showed the best predictive accuracy (AUC = 0.83 ± 0.05) compared to the Ranson (AUC = 0.68 ± 0.06; *P* < 0.02) and Balthazar (AUC = 0.62 ± 0.06; *P* < 0.003). However, there were no significant differences in predicting the duration of treatment for acute pancreatitis (AUC = 0.82 ± 0.05; *P* = 0.53) in comparison with the linear discriminant function. The disadvantage of their ANN model and the entire research was that it developed then tested on the same dataset, so the results will need to be verified by other studies.

Halonen et al. [30] developed two prognostic models to predict potential mortality in pancreatic necrosis [30]. The basis of the research was the retrospective analysis of medical records of 234 patients with severe AP. In the first model, mortality was predicted by the method of logistic regression, in the second, by the ANN. The predictive accuracy of the various models was compared using ROC analysis. The highest predictive accuracy was shown by logistic regression (AUC = 0.862) and the ANN (AUC = 0.847). The rest of the scales demonstrated the following results in predicting mortality in severe AP: Glasgow scale—AUC = 0.536, Ranson—AUC = 0.655, MODS—AUC = 0.781 and APACHE II—AUC = 0.817 [30].

Mofidi et al. [31] developed a neural network model for classifying the severity of AP, predicting organ failure and death. For this study, the authors conducted a retrospective analysis of 664 case histories of patients with AP acute pancreatitis, including 181 patients with severe AP acute pancreatitis (27.3%). As a result, the developed ANN was based on 10 clinical parameters (age, the presence of hypotension, two or more signs of high-resolution survey, the level of PaO_2_, LDH, glucose, urea, calcium, hematocrit, and the number of blood leukocytes) measured initially and then after 48 h. This model showed significantly better results than the APACHE II and Glasgow systems: ANN was more accurate than APACHE II and Glasgow in predicting severe acute pancreatitis (*P* < 0.05 and *P* < 0.01 respectively), in predicting the development of multiple organ failure (*P* < 0.05 and *P* < 0.01) and in predicting a lethal outcome (*P* < 0.05). This work differs from those discussed above by the inclusion of a large number of patients (n = 664), as well as by the fact that development and validation were performed on different groups of patients. Furthermore, all ten input variables are available to the doctor on duty within the first 6 h after hospitalization.

Andersson et al. [32] conducted a study aimed at developing and testing the effectiveness of the ANN model for early prediction of the severity of AP. The authors conducted a retrospective analysis of the results of treatment of 208 patients with AP. The area under the ROC curve for the neural network model was 0.92 (95% CI 0.85–0.99), 0.84 (0.76–0.92) for the logistic regression (*P* = 0.030, *χ*^2^), and 0.63 (0.50–0.76) when assessing the severity of acute pancreatitis using APACHE II (*P* < 0.001, *χ*^2^). The ANN is based on data obtained upon admission of the patient to the hospital. It is sufficiently accurate to predict the severity of AP.

Hong et al. [33] developed the ANN-based DSS for predicting persistent (more than 48 h) organ failure in patients with AP. The sample included 312 patients. The sensitivity of the initial model was 81.3%, specificity—98.9% and accuracy—96.2%. The predictive accuracy of the created ANN (AUC = 0.96 ± 0.02) was statistically significantly better in comparison with the model based on logistic regression (AUC = 0.88 ± 0.03, *P* < 0.001, *χ*^2^) and APACHE II (AUC = 0.83 ± 0.03, *P* < 0.001, *χ*^2^). The authors conclude that the created ANN can be useful for predicting the development of persistent organ failure in patients with AP.

Fei et al. [34] developed an ANN to predict the risk of patients with severe AP developing an acute lung injury. When tested, their ANN showed a sensitivity of 87.5%, specificity of 83.3%, and accuracy of 84.4%. When predicting an acute lung injury, the ANN showed greater accuracy AUC = 0.859 ± 0.048 than logistic regression (AUC = 0.701 ± 0.041). The authors also identified 13 independent variables for predicting an acute lung injury, the most informative among which were the degree of pancreatic necrosis according to CT data, the level of lactate dehydrogenase, and oxyhemoglobin saturation.

### Acute cholecystitis

Eldar et al. [35] used ANN to determine the predictors of conversion from laparoscopic access to laparotomic approach in the acute cholecystitis. Predictive models were selected using conventional statistical methods and ANN methods on the basis of data from 225 patients, who underwent laparoscopic cholecystectomy for acute cholecystitis AC. Direct logistic regression, direct linear discriminant analysis and ANN made it possible to predict the conversion in 0%, 27% and 100% of cases; a negative prediction was given and confirmed in 80%, 85.5% and 97%, respectively. When tested prospectively, the model predicted conversion in 0%, 25% and 67% of cases and non-conversion in 82%, 88% and 94%. According to the authors, a high degree of reliability of prediction reveals the potential of the ANN for allowing a decision to proceed directly to open cholecystectomy without initial laparoscopy.

The ANN was used to predict choledocholithiasis in patients with cholelithiasis and acute cholecystitis. The ANN was developed on the basis of data from 303 patients who underwent surgery for gallstones. The ANN revealed the most informative signs of possible choledocholithiasis were the level of bilirubin, alanine aminotransferase, the diameter of the common bile duct, the number of stones in the gall bladder, the size of the smallest stone, history of biliary colic, history of acute cholecystitis or acute pancreatitis. The authors concluded that ANN is a reliable and user-friendly system that can be successfully used to predict choledocholithiasis [36].

### Upper GI bleeding

Rotondano et al. [37] developed an ANN predicting the probability of death in patients with bleeding from the upper gastrointestinal tract. The ANN was developed and tested on 2380 patients. Their ANN was compared with the Rockall scale [38]. The Rockall scale includes indicators selected by logistic regression—patient age, presence of shock, severity of comorbidities, endoscopic signs of recent bleeding and rebleeding. The developed ANN showed good sensitivity (83.8% versus 71.4%), specificity (97.5% versus 52.0%), accuracy (96.8% versus 52.9%) and AUC (0.95 versus 0.67) [37].

ANN models have also been developed to identify patients with a risk of recurrent bleeding and requiring surgical treatment. These models use clinical, instrumental data and allow identifying such patients with an accuracy of more than 90% [39–43]. Wong et al. [39] developed ANN by retrospective analysis of 22,854 patients with peptic ulcer disease. This ANN was able to identify patients with recurrent ulcer bleeding based on their age, hemoglobin level, localization of the ulcer in the stomach, the presence of other diseases of the gastrointestinal tract, malignant neoplasms and infection. The model identified patients with recurrent ulcer bleeding with AUC of 0.78 and accuracy of 84.3%.

### Perforated peptic ulcers

AI was used to create a model for predicting mortality in patients with perforated peptic ulcers of the stomach or duodenum [44]. Given the complex nature of this disease, which has many nonlinear associations with outcomes, the authors created an ANN to identify risk factors for death. The data of 168 patients were included in the neural network model. The data of 117 patients (70%) were used for the training set, and data of 51 (39%) were used for the test set. The ANN predicted mortality with AUC = 0.90 [95% CI 0.85–0.95], *P* < 0.001.

### Ileus/bowel obstruction

ANN was used to diagnose acute small bowel obstruction based on X-rays. AUC for the developed neural network model was 0.803. AUC increased to 0.971 after additional training. The final ANN had a sensitivity of 91.4% and a specificity of 91.9%. The classification efficiency [45] increases with an increase in the size of the training sample, reaching a plateau for 200 positive training examples.

### Strangulated hernia

Least often, artificial intelligence was involved in solving tactical issues in the treatment of a strangulated hernia. AI elements [46] were used for early recognition of the risk of bowel resection in patients with SH. Bowel resections were performed in 21.0% of 762 patients included in the study (160/762). With the help of ANN, eight factors were identified that are significantly associated with the risk of the need to perform bowel resection in case of its infringement: female gender, age, age > 65 years, femoral hernia, intestinal obstruction, duration of infringement (measured in hours), the number of leukocyte and neutrophilic leukocytes.

## Discussion

In general, a variety of approaches and mathematical algorithms for the construction of decision support systems in medicine have been accumulated and systematized to date. Recently, experts have concluded that the majority of modern and successful models are based on ANN technology, first of all, deep neural networks and deep learning [12]. However, no definitive application has been demonstrated in emergency general surgery. In fact, this field often deals with situations and diseases for which the data accrual and the mathematical model testing is difficult and limited by the circumstances. As a counterpart especially in emergency general surgery, a real need to overcome the limited diagnostic tools requires a definitive improvement in technology and even the introduction of AI tools.

ANN-based decision support systems have been used to diagnose and treat patients with acute surgical pathology over the past 25 years. The first ANN models were mainly developed and intended to support clinical decision making in emergency surgical conditions and differential diagnosis with other diseases that do not require urgent surgery [47]. Subsequently, the main direction of research was to predict the course of the disease at its onset and predict the occurrence of complications. The latest ANNs are aimed at automatic prompting in the diagnosis and prediction of emergency surgical disease without the introduction of data by the surgeon (automated diagnostic methods, for example, analysis of X-ray or MRI images for the automatic detection of pathology, microscopic analysis of biological material, etc.) [48]. The last one will allow the ANN to identify and pay attention to routine pathology independently, reduce the time and cost of examination, and introduce remote diagnostics of emergency surgical conditions [47].

At the same time, all existing ANN-based decision support systems for predicting and diagnosing emergency surgical diseases have certain drawbacks and limitations in their use. Firstly, data from most studies were evaluated retrospectively, which could lead to known biases in results. Secondly, the data were obtained in hospitals of various levels. The last casts doubt on the question of the reproducibility of the method based on data from other clinics. Third, the sample size in most studies was insufficient for developing and testing the ANN (not all studies have trained and tested the ANN on different patient samples). Finally, the solution to the problem of diagnosis and treatment of urgent surgical diseases is strongly associated with the emergence of various interfering factors (confounders), associated mainly with the multi-causality of these diseases, the expressed heterogeneity of the samples of patients with acute surgical diseases. It is usually extremely difficult to assess the degree of various confounding factors impact on the final result [48].

Currently, there are five main limitations of the implementation of decision support systems based on artificial neural networks in medicine and surgery.

First, the quality and reliability of medical information are not always known. The data accumulated in the patient's medical records may be incomplete, contain errors, inaccuracies and non-standard terms. Currently, there are no effective mechanisms for collecting accurate information. Attempts to improve the quality of these analyses often fail due to the complexity of the process. To eliminate this problem, methods for training ANNs on small amounts of reliable information are now proposed [3, 7].

The second significant limitation is the lack of transparency in the decision-making process by the intellectual core of the system. ANN works according to the “black box” principle. If there is an error in the algorithm, and the system made the wrong decision, then it will be extremely difficult to answer the question “why” [3]. Currently, research is underway toward the development of hybrids of the ANN-expert system, which are aimed at improving the understanding of doctors of the way the system makes decisions [7].

The next significant limitation is the selection and development of personnel capable of effectively using and maintaining intelligent systems [8].

The fourth limitation is a high cost of projects, which is associated with the need to configure the new system for the data accumulated in a particular medical institution and to form a qualified and motivated team [9].

One of the most important limitations is that data sets have to be taken outside a medical institution, and this threatens the security of storage. It is no coincidence that many projects of the introduction of AI were stopped because of the risks related specifically to information security [7].

Despite the existing problems, researchers see the further use of neural networks in software that will quickly and accurately process large amounts of data. This will ultimately lay the foundation for high-performance medicine, which will be based on big data and reduce dependence on human resources [5]. The need for quick decision-making in emergency surgery when there is a limited resource is especially important. It will require the development of simple and accurate decision support systems based on AI [6, 48, 49].

Currently, the term Surgery 3.0 [6] has appeared. It characterizes modern changes in surgery by analogy with the Internet. The Internet is evolving from a collection of passive readers (Web 1.0) to a modern, interactive, AI-powered audience (Web 3.0). Surgery will change radically with the development of computational science and AI toward surgical artificial intelligence (Surgery 3.0) in order to improve the results of treatment of surgical patients [6].

However, artificial intelligence is currently one of the most controversial issues in the world. Written AI algorithms can contain errors that can lead to unintended consequences and unfair results. AI researchers must consider the need to prevent ethical violations [50].

## Conclusion

The development and implementation of a DSS based on ANNs is a promising direction for improving the diagnosis and treatment of emergency surgical condition and their complications. At the same time, further improvement of the ANN is necessary, taking into account the shortcomings of previous studies. Developed DSS in medicine in general and, especially, in emergency surgery should be simple, accurate and as close as possible to the doctor's workplace.

## Data Availability

Not applicable.

## Abbreviations

DSS: Decision support systems
ANN: Artificial neural network
AI: Artificial intelligence
AA: Acute appendicitis
AP: Acute pancreatitis
APACHE II: Acute physiology and chronic health evaluation
SPS: Severity of physiological state
AC: Acute cholecystitis
AUC: Area under curve
ROC: Receiver operating characteristic
CI: Confidence interval
LOS: Length of stay
MODS: Multiple organ dysfunction score
LDH: Lactate dehydrogenase
